# Comparative phylogenomics and multi-gene cluster analyses of the Citrus Huanglongbing (HLB)-associated bacterium *Candidatus *Liberibacter

**DOI:** 10.1186/1756-0500-1-72

**Published:** 2008-08-28

**Authors:** Harshavardhan Doddapaneni, Huihong Liao, Hong Lin, Xianjin Bai, Xiaolong Zhao, Edwin L Civerolo, Michael Irey, Helvecio Coletta-Filho, Gerhard Pietersen

**Affiliations:** 1 USDA-ARS. San Joaquin Valley Agricultural Science Center, 9611 So. Riverbend Avenue, Parlier, CA 93648, USA; 2 University of California Davis, Department of Viticulture and Enology, Davis, CA 95616, USA; 3 Guangxi Academy of Agricultural Sciences, Nanning, PR China; 4 Agricultural College, Guangxi University, Nanning, 530004, PR China; 5 Guangxi Citrus Research Institute, Guilin, PR China; 6 United States Sugar Corporation, Clewiston, Fl 33440, USA; 7 Instituto Agronémico de Campinas, Cordeirópolis, Brazil; 8 Citrus Research International c/o University of Pretoria, Pretoria, Republic of South Africa

## Abstract

**Background:**

Huanglongbing (HLB, previously known as citrus greening), is associated with *Candidatus *Liberibacter species and is a serious threat to citrus production world-wide. The pathogen is a Gram negative, unculturable, phloem-limited bacterium with limited known genomic information. Expanding the genetic knowledge of this organism may provide better understanding of the pathogen and possibly develop effective strategies for control and management of HLB.

**Results:**

Here, we report cloning and characterization of an additional 14.7 Kb of new genomic sequences from three different genomic regions of the *Candidatus *Liberibacter asiaticus (Las). Sequence variation analyses among the available *Ca*. Liberibacter species sequences as well as the newly cloned 1.5 Kb of *rpo*B gene from different *Ca*. Liberibacter strains have identified INDELs and SNPs. Phylogenetic analysis of the deduced protein sequences from the cloned regions characterizes the HLB-associated *Candidatus *Liberibacter as a new clade in the sub-division of the α-proteobacteria.

**Conclusion:**

Comparative analyses of the cloned gene regions of *Candidatus *Liberibacter with members of the order Rhizobiales suggest overall gene structure and order conservation, albeit with minor variations including gene decay due to the identified pseudogenes. The newly cloned gene regions contribute to our understanding of the molecular aspects of genomic evolution of *Ca*. Liberibacter.

## Background

Huanglongbing (HLB) previously known as citrus greening, associated with *Candidatus *Liberibacter species is the most serious threat to citrus production due to reduced fruit quality and tree death [1]. Currently, three major forms of the disease are recognized as being associated with three different *Candidatus *Liberibacter species, *Ca*. Liberibacter asiaticus (Las), *Ca*. Liberibacter africanus (Laf), and *Ca*. Liberibacter americanus (Lam) [2]. The bacteria associated with HLB are Gram negative, unculturable, phloem-restricted α-proteobacteria and are transmitted by psyllids [1,3]. Until recently, the available genetic information included the 16S rRNA, the *rpl*KAJL-*rpo*BC operon and the OMP regions [4-9]. Collectively, this amounts to ~15.6 Kb of the non-redundant DNA sequences from these three regions of the genome. In our recent report, we cloned and characterized 8.56 Kb of genomic sequences from a Las strain using a genomic walking method [10].

Studies of the comparative gene organization and gene order among related bacteria, can lead to improved understanding of the functional significance of gene arrangements among them [11,12]. Such information can either be derived from phylogenetic profiles [13] or from comparative genome analyses [14]. The information may also provide insight into these organisms' evolutionary history and metabolic capabilities [15].

In this study, we report cloning and characterization of an additional 14.7 Kb of new genomic DNA from Las following the recently described modified genomic walking method [10]. We further discuss the comparative phylogenomics of *Ca*. Liberibacter species gene structure and evolution to understand the gene organization of this obligate plant pathogen.

## Results

### Cloning of new genomic regions, gene characteristics and homology studies

Primers were designed based on the previous sequence information and incorporated into the walking strategy as detailed earlier [10]. Using the sequence data from the genomic walk and primers listed in Table 1, genomic DNA fragments were amplified as PCR products directly from the DNA extracts of infected tissue. For details, see Additional file 1.

**Table 1:** Confirmation primers designed and directly re-amplified from HLB-infected samples.

**Target region**	**Forward primers (5'-3')**	**Reverse Primers (5'-3')**	**Amplicon ** size (bp)
**Region-1**			
	Region-1-L-F GGTCAATTCAATGCGCTATAC	Region-1-L-R CAGGAATCTGTCGAGCAATGG	3,308
	Region-1-R-F1 GGCATGTGTTGGTCTTGGAA	Region-1-R-R1 TCGGTGATTCAATAATTTCC	3,019
	Region-1-R-F 2 CTCAATGGTGGCAGTTCGCTG	Region-1-R-R2 TTCTTTCCCAAGACCAACACATGC	398
RpoB Las primer	RpoB-las-F AATTTTTCTGTTCCTCGCAGC	RpoB-las-R CAGCGAACTGCCACCATTGAG	1,530
RpoB Las & Laf common primer pair	RpoB-las-laf-F TCAACTTGAAGAACATGTGAACTCTCTTTCGC	RpoB-las-laf-R CAGCGAACTGCCACCATTGAG	1,508
**Region-2**			
	Region-2-L-F GCGGGCCTGTTGATAATCCTGC	Region-2-L-R CACATAACCAAGTCAACCCA	1,931
	Region-2-R-F CAACAACCCTGACCTCCATC	Region-2-R-R CACAGTTTCATAGCCTCCCA	1,041
**Region-3**			
	Region-3-L-F GCAGCTGGAAGGGGATTCAC	Region-3-L-R GATAGCACCCTGATATTACACAA	4,150
	Region-3-R-F GAGGAACCGTTGAGTATGGC	Region-3-R-R TTTCTACAGTCTACGATGCG	1,124

Primers were designed based on the DNA sequences obtained by a genomic walking protocol. Amplification parameters are detailed in the Methods section.

#### Region-1

This region was extended by 3,218 bp at the 5' end and by 2,673 bp on the 3' end to the previously reported sequence [10]. With this newly cloned sequence and the previously known sequence, there is 17.1 Kb of DNA sequence known from this region. BLAST based similarity searches of the 5' end identified a partial gene coding for fimbrial assembly protein PilP, and a full-length gene coding for phosphoserine aminotransferase (*serA*). However, there is a 950 bp DNA sequence between this gene and the previously identified pseudogene D-3-phosphoglycerate dehydrogenase, where no gene models were identifiable. Partial gene sequence (2.3 Kb) of the *rpo*C gene was identified at the 3' end. In total there are now 12 genes and one pseudogene in this region.

#### Region-2

In this study, the 5' end of region-2 was extended by 1,776 bp and the 3' end by 1,908 bp. This brings the DNA sequence known in this region of the genome to 6.6 Kb. Importantly, we have now obtained the full-length sequences of the 16S region, as BLAST searches have identified a pseudogene caused by a frame shift mutation next to the 16S rRNA gene suggesting that we have walked out of the 16S rRNA gene region. At the 3' end, we cloned 1,908 bp of new DNA sequence of the 23S rRNA gene.

#### Region-3

This region was extended beyond previously reported  [10] to a further 4,150 bp at the 5' end and by 1,037 bp of DNA sequence at the 3' end. With this new DNA sequence, the sequence for the *omp *gene region known from this organism is now 10.5 Kb long. BLAST similarity searches have identified a partial gene coding for putative transmembrane protein (*rps*B) at the 5' end, and four full-length genes coding for proteins and a pseudogene, including elongation factor Ts (*tsf*), uridylate kinase (*pyr*H), ribosome recycling factor (*frr*), frame shift induced pseudogene for undecaprenyl pyrophosphate synthetase (*upp*S), and phosphatidate cytidylyltransferase (*cds*A2). Similarly, at the 3' end, the remaining sequence of the gene coding for the full-length lipid A biosynthesis acyl- [acyl-carrier-protein]-UDP-N-acetylglucosamine O-acyltransferase was cloned, as well as the partial DNA sequence coding for phosphatidate cytidyltransferase gene (COG3494). In total, there are now 11 genes and a pseudogene known in this region.

### Inter-strain; inter-species sequence variations

At the time when this study was conducted, a total of 86 *Ca*. Liberibacter sequences were publicly available in the GenBank databases. These sequences from Las, Laf and Lam strains were compared to identify sequence variations within and among the three genomic regions to define the cumulative diversity. Of these 86 submissions, 76 are Las sequences, seven are Laf sequences and three are Lam sequences. The majority of the submissions (58 of the 86) were from the 16S-23S region with the rest of the sequences were from gene cluster (19 sequences) and *omp *(nine sequences) regions. The variations in the 16S rRNA gene sequence among the three *Ca*. Liberibacter species ranged from 90–94%. There is a large INDEL of ~17 bp identified in the Lam strain compared to the Las and Laf strains in this region. A sequence variation of 29% was noted for the *omp *gene sequences between Las and Laf strains.

Intra-species sequence conservation for 16S-23S region among the Las and Lam strains was higher (>95%) than the two Laf strains (93%). Similarly, the six Las strains showed little variation (<1%) for the *omp *gene.

The primer pair RpoB-las-laf-F and RpoB-las-laf-R amplified a 1,508 bp fragment from the DNA extracts from citrus leaf tissues infected with multiple strains of Las strain from China, Japan, Sao Paulo (Brazil) and Florida (USA), as well as the Laf strain (Mpumalanga) from South Africa (Tables 1 &2). The DNA fragments associated with Las strains from Florida and Brazil were identical for the above region. In contrast, there were two SNPs at nucleotide positions 930 and 1,392 in these strains and the strain from China. Both of these SNPs are transversion (T to C) mutations. The Laf strain differed by 180 SNPs and 182 SNPs from the Florida and the Brazilian Las and Chinese strain, respectively.

**Table 2:** PCR amplification results targeting primer pair 'RpoB-las-laf-F and RpoB-las-laf-R' for the 1.5 Kb DNA fragment of the *rpo*B gene.

**Species**	**Sample ID**	**PCR**	**County/State/Country**
***Ca*. Liberibacter asiaticus**	10002, CCLP Crows Nest	-ve	Hendry, Florida, USA
	10795, Barron Collier Oak Hammock	+ve	Hendry, Florida, USA
	10938, Orange Co. Joshua	+ve	Desoto, Florida, USA
	11206, USDA Fort Pierce	+ve	St. Lucie, Florida, USA
	11869, Lykes Lake Placid	+ve	Highlands, Florida, USA
	1-Bra- 327,	+ve	Sao Paulo, Brazil
	5-Bra- 40,	+ve	Sao Paulo, Brazil
	6-Bra- 87,	+ve	Sao Paulo, Brazil
	11-Bra- 319,	+ve	Sao Paulo, Brazil
	10-CHN	+ve	Guangxi province in China
	16-CHN	+ve	Guangxi province in China
	17-CHN	+ve	Guangxi province in China
	18-CHN	+ve	Guangxi province in China
	19-CHN	+ve	Guangxi province in China
	6-MD	+ve	Japan
***Ca*. Liberibacter africanus**	333, Eureka Lemon	+ve	Caledon, Western cape, South Africa
	UPCRI 05-0232, MCE Marsh	+ve	Mpumalanga, South Africa
	UPCRI 05-0252, Navalina	+ve	Mpumalanga, South Africa
	UPCRI 06-0026, ITSC Museum tree	+ve	Mpumalanga, South Africa
	UPCRI 06-0071, M 45/06	+ve	Mpumalanga, South Africa
***Ca*. Liberibacter americanus**	39-Bra- 275	-ve	Sao Paulo, Brazil
	48-Bra- 304	-ve	Sao Paulo, Brazil

### Phylogenetic analysis

**Figure 1:**
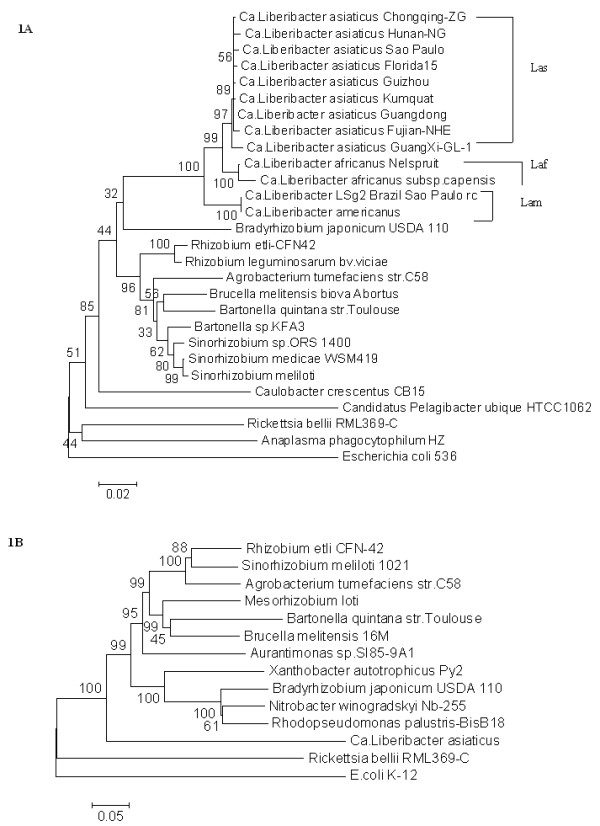
**A-B. Phylogenetic analyses of the cloned genes of *Ca*. Liberibacter asiaticus**. Neighbor-joining trees were generated using MEGA 4 software with 1,000 bootstrap replications. 1A (16S rRNA) and 1B Concatenated tree of eight proteins as described in the Methods section. For both the trees, *Escherichia coli *was added as the outgroup.

Topology of the 16S rRNA tree for nine *Ca*. Liberibacter asiaticus strains, two *Ca*. Liberibacter africanus strains and two *Ca*. Liberibacter africanus strains segregated according to the existing species classification (Fig. 1A). Interestingly, for the *Ca*. Liberibacter asiaticus, the strains from Florida, USA and Sao Paulo, Brazil were interspersed with multiple strains from China suggesting the lack of geographic grouping within *Ca*. Liberibacter asiaticus. Among the three species, only the *Ca*. Liberibacter africanus strains showed longer inner branches owing to their sub-species status. With respect to the other bacteria used in the analyses, the 16S rRNA Neighbor-joining tree and the concatenated protein sequence (3401 aa) derived tree for *Ca*. Liberibacter asiaticus showed similar tree topology (Fig. 1A–B). Both the trees placed *Ca*. Liberibacter species at the bottom of the order Rhizobiales with a high bootstrap value of 96–100. The tree topology suggests that *Ca*. Liberibacter's segregated earlier from the Rhizobiales and Rickettsiales in the α-proteobacterial division, suggesting its independent evolution as a sub-division.

### Gene organization and gene order conservation

#### Region-1

**Figure 2:**
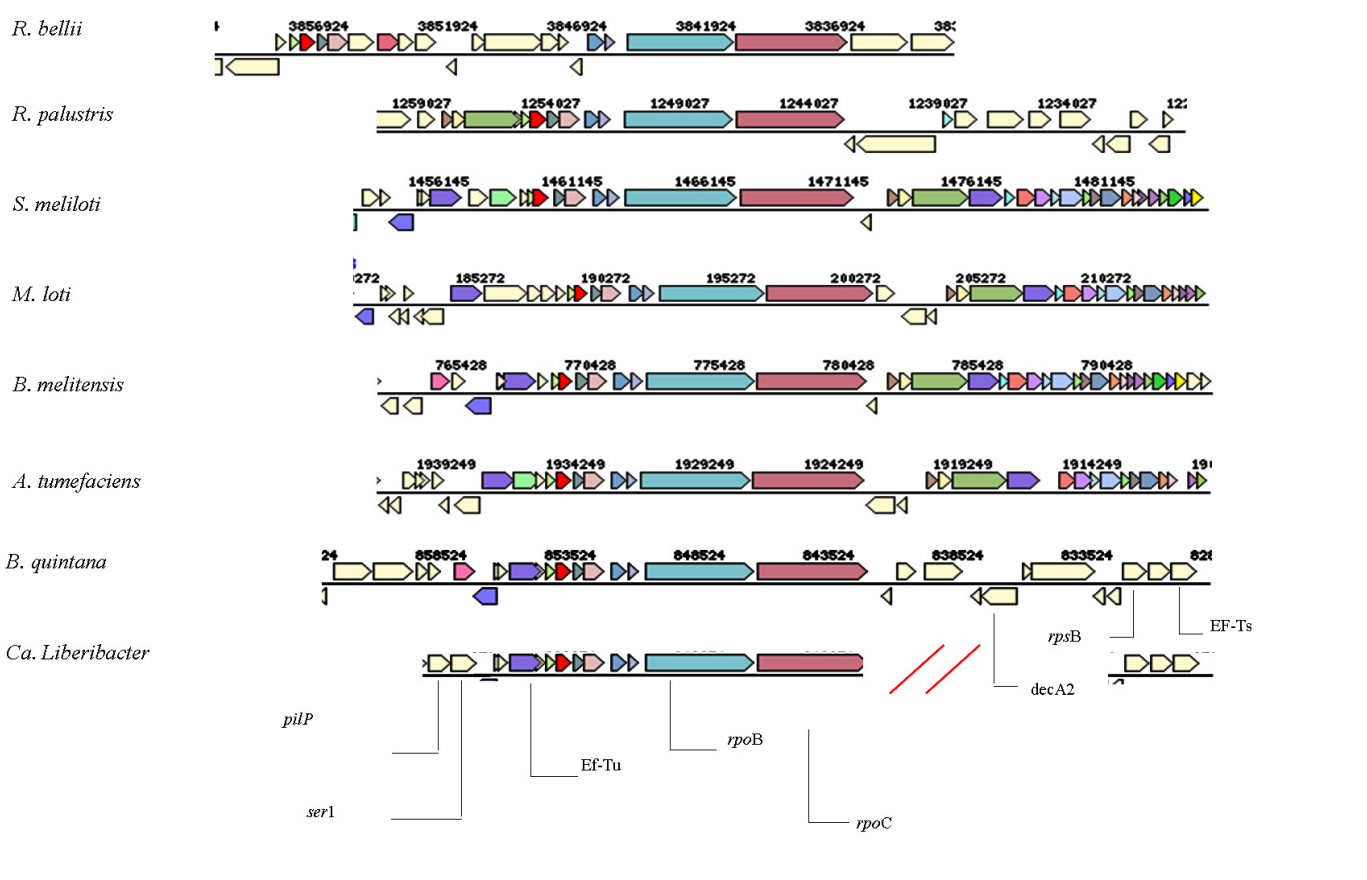
**Comparative gene cluster region (Region-1) organization**. Genes denoted by the same color (except light yellow) are from the same orthologous group (top COG hit). Light yellow = no COG assignment. Figures 2-4 were generated at the IMG website as detailed in the Methods section (Additional file 1). The corresponding matching genomic positions were 1451145–1471145 for *S. meliloti*; 856122-8543524 for *B. quintana*; 760428–780428 for *B. melitensis*; 1745952–1758326 for *R. etli*; 3861924-3836924 for *R. bellii*; 1259027–1244027 for *R. palustris*; 180272–200272 for *M. loti *and 1936179–1931230 for *A. tumefaciens*. Between *sec*E and Ef-Tu proteins, the three plant symbiotic bacteria have a NDP-sugar epimerase protein (putative oxidoreductase protein; NP_385447 *S. meliloti*) which is absent in the other three bacteria. Further, only the Las strain has the tRNA-meth on the opposite strand, while other bacteria (*B. quintana, B. melitensis, R. palustris, M. loti, S. meliloti*) encode the tRNA/rRNA methyltransferase protein. This gene, however, was different from the cloned tRNA-meth (*trm*U) Las gene. On the other hand, there is a membrane protein coding gene (*ter*C) in *A. tumefacians *at that position. Similarly, none of the above bacterial species have the genes D-3-phosphoglycerate dehydrogenase and the phosphoserine aminotransferase in the gene cluster region that is present in *Ca*. Liberibacter asiaticus. The (**//**) indicates that this region is not yet cloned.

Comparison of the gene order in the Region-1 from nine bacterial species including *Ca*. Liberibacter species suggests conservation among these bacteria from *tsf *(Ef-Tu) to the *rpo*C gene for this region with minor variations (Fig. 2; *R. etli *figure not included).

#### Region-2

**Figure 3:**
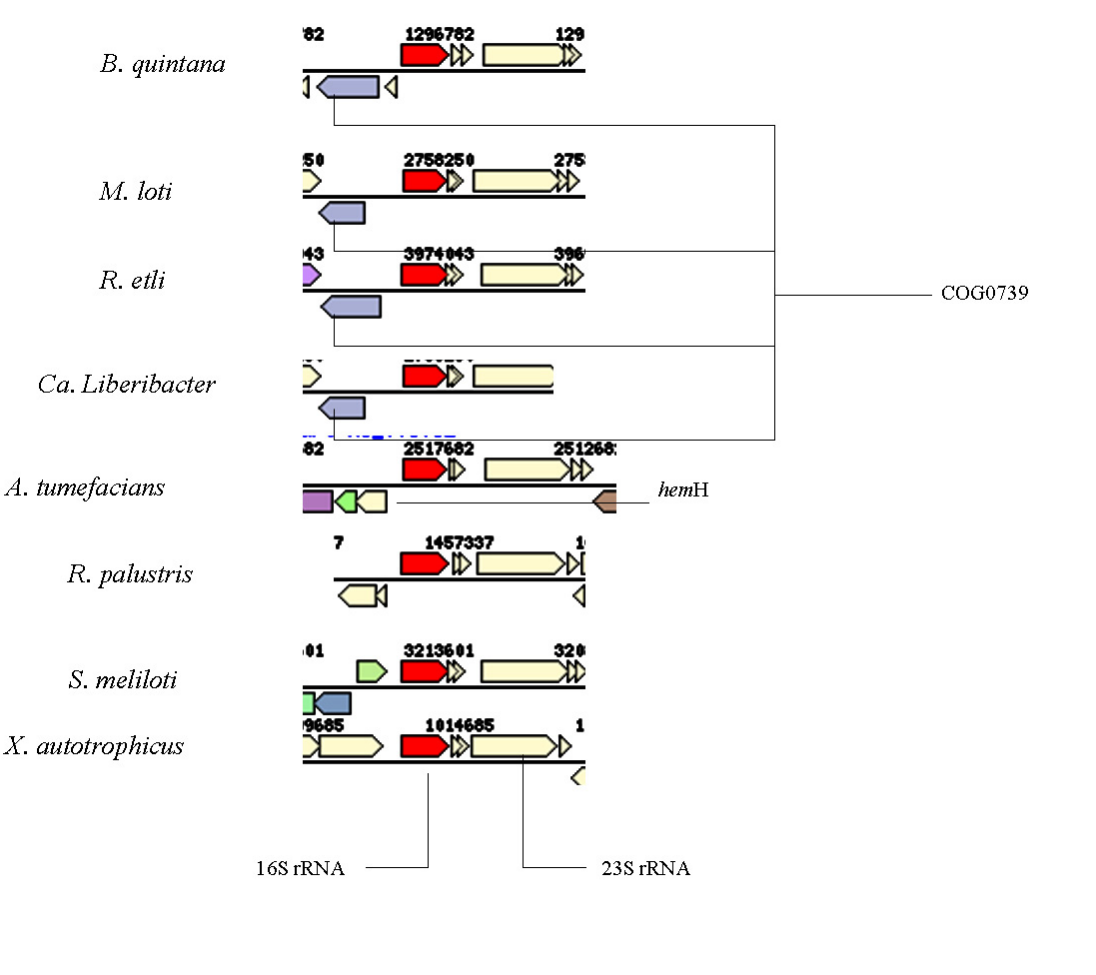
**Comparative 16S-23S rRNA region (Region-2) organization**. Genes denoted by the same color (except 23S rRNA gene which is colored in light yellow) are from the same orthologous group (top COG hit). Light yellow = no COG assignment for other genes. The 16S gene is denoted in red. Explanation of terms: *hem*H- gene coding for phosphoribosylaminoimidazole-succinocarboxamide synthase protein; COG0739-putative membrane proteins related to metalloendopeptidases. The genomic regions matching that found in the Las cloned sequence was from genomic position 3213601 for *S. meliloti*; 1296782 for *B. quintana; *198643 for *B. melitensis*; 3974043 for *R. etli*; 2758250 for *M. loti*; 1014685 in *X. autotrophicus*; 1457337 for *R. palustris*, and from 2517682 of the *A. tumefaciens *genomes. In addition, there are two additional coding sequences on the reverse strand in *A. tumefaciens*, one of which belongs to the cupredoxin family.

The gene order of Region-2 was 16S rRNA-tRNA^Ile^-tRNA^Ala^-23S rRNA and was relatively conserved among these eight bacterial species with minor variations especially for the genes cloned from the 5' end (Fig. 3).

#### Region-3

**Figure 4:**
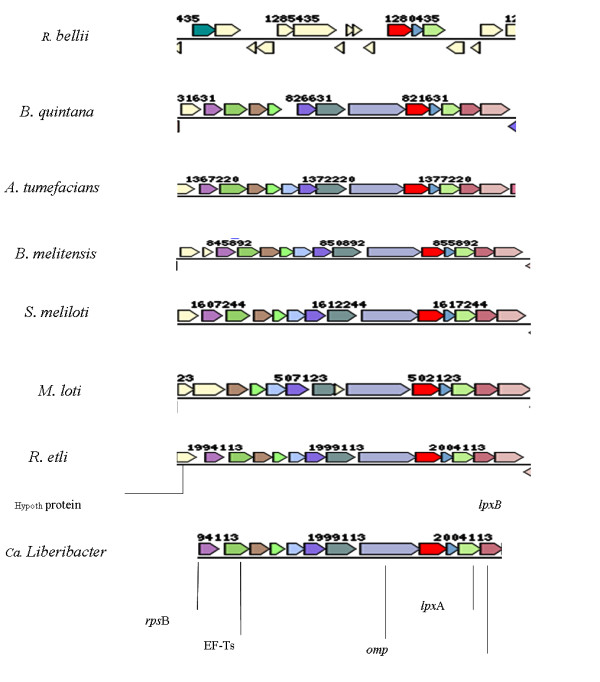
**Outer membrane protein region (Region-3) comparative organization**. Genes of the same color (except light yellow) are from the same orthologous group (top COG hit). Light yellow = no COG assignment. The cloned Las *omp *region gene cluster matches the other bacterial genomic sequences at the following genome co-ordinates: *S. meliloti *(1607244–1622244); *B. quintana *(831631-816631);*B. melitensis *(845892–860892); *R. etli *(1994113–2009118); *A*. *tumefaciens *(1367220–1387220); *M. loti *(512123-492123) and *R. bellii *(1280435).

An overall similarity in gene structure conservation was observed for the *omp *region where the gene order of all the 11 genes (*rps*B**-***tsf-Tu***-***pyr*H**-***frr***-***upp*S**-***cds*A2**-***yae*L**-***omp***-***lpx*D**-***fab*Z**-***lpx*A) was conserved in seven bacteria (*S. meliloti, B. quintana, B. melitensis*, *R. etli, A. tumefaciens, M. loti *and *Ca*. L. asiaticus (Fig. 4). In these bacteria, there was another conserved gene of the COG03494 group (uncharacterized conserved protein), similar to phosphatidate cytidylyltransferase, at the 3'-end of this 11 gene cluster. In the other six bacteria, *M. loti*, *S. meliloti*, *B. melitensis*, A.*tumefaciens B. quintana *and *R. etli*, this gene is followed by *lpx*A gene. This region was least conserved in the bacterium *R. bellii*.

## Discussion

In this study, we report cloning and characterization of new genomic regions from *Ca*. Liberibacter species and compare the overall sequence diversity in the sequences of these bacteria in the GenBank.

Comparison of DNA sequences for the 16S, *omp *and *rpo*B genes from GenBank and the sequences cloned in this study shows that there is very little sequence variation among the different *Ca*. Liberibacter species suggesting strong host and/or environmental selection and a genetically stable lineage of the pathogen. The 16S sequences were more conserved among the Las strains while; a slightly higher degree of sequence variation was noted for the Laf strains. Alignments for a ~1.5 Kb region of the *rpo*B of Las strains and Laf strains revealed that strain from China differed by two SNPs from the Japan, Florida and Brazil strains, which were identical at this locus. These two SNPs were possibly introduced later in the Chinese strain after their separation from the other Las strains.

Our phylogenetic analyses based on the 16S rRNA and the concatenated protein sequences from eight genes, places *Ca*. Liberibacter species as a new clade in the sub-division of the α-proteobacteria [2]. This agrees with the previously reported 16S rRNA and *omp *gene based phylogenetic analyses [16,17]. The inclusion of the new genes showed similar results to those based upon the 16S rRNA and the *omp *gene, suggesting future inclusion of these genes along with the 16S rRNA and other *omp *genes should enhance understanding of *Ca*. Liberibacter strain diversity studies, especially in situations where the other two conserved genes fail to differentiate the strains. Our results also suggest that *Ca*. Liberibacter evolved along with the members of the order Rhizobiales and Rhodobacteriales after the separation of the order Rickettsiales, but branched out before the expansion of the order Rhizobiales. The comparative genomic analysis of eight of these bacteria based on the three genomic loci cloned from the *Ca*. Liberibacter asiaticus revealed overall gene order and operon conservation with some notable differences. Especially, the selective incorporation/retention of a NDP-sugar epimerase protein in plant associated bacteria (*A. tumefaciens, S. meliloti *and *R. etli*), and presence of pseudogene for D-phosphoglycerate dehydrogenase gene (*serA*) and the two other identified pseudogenes could indicate ongoing host adaptation. The *serA *gene is also absent in the other phloem limited bacterium *Buchnera aphidicola *that lives in aphid gut, which feed on phloem sap, indicating that *Ca*. Liberibacter might survive on the plant-derived serine by direct intake from phloem sap.

## Conclusion

The genomic regions cloned in this study have provided new information for better understanding molecular aspects of genomic evolution of *Ca*. Liberibacter and taxonomically related bacteria.

## Authors' contributions

H. Liao, HD, and H. Lin cloned the sequences. HD did the comparative genomic analyses wrote the manuscript and along with H. Lin and ELC did the data interpretation. XB, XZ, MI, HC and GP collected infected citrus samples, did DNA extractions and HLB conformation tests. All the authors have read, commented and approved the final manuscript.
